# The epistemology of modality and the problem of modal epistemic friction

**DOI:** 10.1007/s11229-018-1860-2

**Published:** 2018-07-09

**Authors:** Anand Jayprakash Vaidya, Michael Wallner

**Affiliations:** 1 Department of Philosophy, San Jose State University, 1 Washington Square, San Jose, CA 95192 USA; 2 Department of Philosophy, University of Graz, Heinrichstraße 26, 8010 Graz, Austria

**Keywords:** Modality, Essence, Conceivability, Counterfactual reasoning, Deduction

## Abstract

There are three theories in the epistemology of modality that have received sustained attention over the past 20 years (1998–2018): conceivability-theory, counterfactual-theory, and deduction-theory. In this paper we argue that all three face what we call *the problem of modal epistemic friction* (PMEF). One consequence of the problem is that for any of the three accounts to yield *modal knowledge*, the account must provide an epistemology of essence. We discuss an attempt to fend off the problem within the context of the internalism versus externalism debate about epistemic justification. We then investigate the effects that the PMEF has on reductive and non-reductive theories of the relation between essence and modality.

## Introduction

The folk question at the heart of the epistemology of modality is: *how can we come to know what is possible?* One important project within philosophy is the refinement of folk questions into theoretical questions. The *access question* refines the folk question by asking: how is it that we gain access to or acquire epistemic standing for beliefs about modality, such as that it is possible for *x* to be F? The *navigation question* refines the folk question by asking: how we can reason with justification from one kind of modality to another, say from logical to metaphysical to physical modality?^1^ Within the epistemology of modality one finds a number of answers to these questions.

- Intuition [Bonjour (1998), Bealer (2002), Chudnoff (2013)]
- Conceivability [Yablo (1993), Chalmers (2002)]
- Counterfactuals [Williamson (2007), Kroedel (2012)]
- Deduction [Kripke (1971), Lowe (2008, 2012), Hale (2013)]
- Conventions [Sidelle (1989), Sveinsdóttir (2008, 2013), Thomasson (2013)]
- Implicit Knowledge of Principles of Possibility [Peacocke (1998)]
- Imagination [Hume (1980), Gregory (2004), Kung (2010), Ichikawa and Jarvis (2011)]
- Free Variation [Husserl (1973), Mohanty (1991), Tieszen (2005)]
- Perception [Legg (2012), Legg and Franklin (2017), Hanrahan (2009), Strohminger (2015)]
- Similarity Reasoning [Roca-Royes (2017), Hawke (2011)], and
- Theories or Arguments [Fischer (2016a), Bueno and Shalkowski (2014)].

Some of the answers above can usefully be classified through the use of Hale’s (2002) distinction between necessity-first and possibility-first approaches to the acquisition of modal knowledge. The overarching idea of Hale’s distinction is that the architecture of modal knowledge might be asymmetric, with one of either necessity or possibility being dominant and the other being recessive. We can mark the difference between the two approaches as follows.

(N): Knowing that *p* is possible involves determining that there are **no conflicting necessities** that conflict with *p*’s being possible
(P): Knowing that *p* is necessary involves determining that there are **no conflicting possibilities** that conflict with *p*’s being necessary

On the necessity-first approach, (N), the acquisition of modal knowledge is primarily a function of coming to know necessities. Necessity is dominant, and possibility is recessive. On the possibility-first approach, (P), the acquisition of modal knowledge is primarily a function of coming to know possibilities. Possibility is dominant, necessity is recessive.^2^ In other words, we either come to know necessities and infer possibilities on that basis, or we come to know possibilities and infer necessities on that basis.

Recently, there has been a push to go beyond rationalism about modal knowledge, where rationalism is primarily identified as being pursued by Peacocke (1998), Chalmers (2002), Bealer (2002), and Lowe (2008, 2012). Fischer and Leon’s (2017), *Modal Epistemology After Rationalism*, is an outstanding exploration of the prospects of modal empiricism over varieties of rationalism. Some of the works in this anthology favor the possibility-first approach, such as Roca-Royes’ (2017) similarity-based approach. Possibility-first approaches are often tied to an empiricist program in the epistemology of modality, although they need not be. While we are very sympathetic to the possibility-first approach, and in favor of the recent growth in exploring empiricism as opposed to rationalism, our own view is that there is a need for more debate between necessity-first and possibility-first approaches directly. In particular we are interested in exploring the prospects for what we call the *Hale*-*Branch* of the necessity-first approach.

It is important to note that Fischer (2016b) offers an important response to Hale’s defense of the necessity-first approach to the architecture of modal knowledge. Here we will not offer a direct response to Fischer’s argument. Rather, we will pursue an exploration of the *Hale*-*Branch*, as a way of pushing forward the view that the best approach on the necessity-first side of the debate might turn out to be a variant of Hale’s own essence-based approach. If our argument is successful, we take it that the debate that needs a hearing is one between variants of the *Hale*-*Branch* and variants of possibility-first approaches, such as Roca-Royes’ similarity-based approach.

This debate would be set against what we think has already had a sound hearing: debates over mental-operation accounts, such as conceivability-theory, counterfactual-theory, or deduction-theory.^3^ In short, we believe there needs to be more debate between essence-first and possibility-first approaches to the epistemology of modality. As a consequence, our choice of theories to engage can be seen as a function of our goal: the development of *the problem of modal epistemic friction*, which we argue is a major problem for conceivability-, counterfactual-, and deduction-theories. While we offer no defense of the claim that the *Hale*-*Branch* of the epistemology of modality is the most successful account on offer, we do think that it has the correct ingredients for being the best way to think about the necessity-first approach to the epistemology of modality. Thus, there could be a direct debate about the architecture of modal knowledge between essence-first and possibility-first approaches.

Our plan for the paper is the following: In Sects. 2–4 we develop the problem of modal epistemic friction (PMEF) through an examination of conceivability-, counterfactual-, and deduction-theories. In Sect. 2 we articulate the PMEF within the confines of one of the oldest theories in the epistemology of modality: conceivability-theory. In Sects. 3 and 4 we go on to make the problem precise as we explore it within the context of counterfactual- and deduction-theory. In Sect. 5 we summarize the problem and argue that the PMEF demands that the epistemology of modality be supplemented by an epistemology of *essence*.^4^ In Sect. 6 we discuss a specific attempt to fend off the PMEF within the context of Williamson’s (2007) *counterfactual*-*theory* that relies on the debate between internalism and externalism about justification. In Sect. 7 we explore the debate between *reductive* and *non*-*reductive* interpretations of essentialism. We observe that the PMEF has a different effect on either of these interpretations. We end by investigating Hale’s argument for *Non*-*Reductive Finean Essentialism* (NRFE). In Sect. 8 we offer some concluding remarks.

## Conceivability-theory

Specific theories of conceivability, such as Yablo (1993) and Chalmers (2002), vary in interesting ways because of the different semantical, metaphysical, and epistemic ways in which the notion of *conceivability* can be filled out. However, one can examine the general structure of conceivability-theory and what problems the general structure faces by taking note of three important connections that are usually established by specific theories in different ways. Where we read ‘*ϕ* ⇒* ψ*’ to mean ‘we are epistemically led from *ϕ* to *ψ*, and ‘*x*’ is a variable ranging over various kinds of modality, such as logical, epistemic, metaphysical, or physical, a conceivability-theory typically assumes (MEQ) and says something robust about (CON) and (INC).

(MEQ): ¬◊^*x*^ ¬P ≡_eq_ □^*x*^P
  It is necessary in sense *x* that P iff it is not possible in sense *x* that P is false.
(CON): ◊^*c*^P ⇒ ◊^*x*^P
  We are *epistemically* led from the conceivability of P to the belief that P is possible in sense *x*.^5^
(INC): ¬◊^*c*^P ⇒ ¬◊^*x*^P
  We are *epistemically* led from the inconceivability of P to the belief that P is impossible in sense *x*.

Vaidya (2017) discusses a number of critical questions that conceivability-theory faces. One of these questions, referred to here as the *input question*, asks: to what degree does conceivability depend on its inputs, the conceptions/representations we are working with when we conceive of a scenario or situation? To get a firm grip on the force of this question we will examine the case of *whether* and *how* conceiving of a scenario in which transparent iron is present *could* guide one to a sound judgment that *it is possible for transparent iron to exist*. The presentation of the case of transparent iron against the backdrop of the input question will be used to make a first pass formulation of the PMEF.

For a subject *x* to conceive of a scenario *S* in which transparent iron is present, it must be the case that *x* constructs a representation *R* of *S* through some mode of construction, such as purely through the assembly of linguistic items or images, or through a hybrid combination of linguistic and imagistic items. These options simply exhaust the possibilities for how a subject can construct a representation of a scenario of the kind we are considering. Now once the (alleged) representation *R* of the scenario *S* is constructed, for *x* to be *confident* that *R* is indeed a representation of *S*, *x* should be *confident* that the *essence* of iron does *not* keep *R* from containing transparent iron. That is, *x* should be confident that *R* verifies that transparent iron is present only if *R* involves no violation of what it is for something to be an instance of the kind iron.^6^ For *x* to be confident that such a violation has not occurred, it would seem that *x* must have either implicit or explicit essentialist information about *what* iron is. This essentialist information must also put *x* in a position to know that transparent iron is possible on the basis of conceiving *S* either through linguistic items, images, or a hybrid combination of them.

In opposition to this claim one might think that they can conceive of transparent iron by pictorially conceiving of a bar of some transparent material while assuming or pretending that this material is iron. That such a strategy leads us to the genuine possibility of transparent iron can be doubted by looking at the following analogy with a well-known example in the epistemology of modality: the case of conceiving of a proof of Goldbach’s conjecture (GC). As van Inwagen (1998) points out and Chalmers (2002) discusses, conceiving of a scenario in which mathematicians have announced a proof of Goldbach’s conjecture *is not a sufficient proof* for the possibility of Goldbach’s conjecture. Using this point, we can make the following analogical argument. Conceiving of a scenario in which mathematicians have announced that GC is true is *not* sufficient for proving that GC is possibly true. Conceiving of a scenario in which a bar of some transparent material is stipulated to be transparent iron is *epistemically akin* to conceiving that mathematicians have announced that GC is true. Therefore, conceiving of a scenario in which a bar of some transparent material is stipulated to be transparent iron is *not* sufficient for proving the claim that it is possible for transparent iron to exist. The problem is simply that *the relevant depth of detail* has not been conceived of in either scenario.

To summarize: In considering the case of *transparent iron* we come to see that we need information about what an entity is essentially in order to determine whether a given representation *R* is indeed a representation of a (genuinely possible) scenario *S* and whether *R* is indeed coherently constructed so as to reliably guide us to a substantive modality, such as metaphysical modality. Our point is that we should only be confident that we have conceived of a genuinely possible scenario *S*, that our conceivability exercises track a genuine possibility, if the representation of *S* has been constructed in a way that is consistent with the kinds of things involved in *S*.

Thus, our first pass presentation of the PMEF arises in relation to conceivability-theory with respect to the question: *when should we be confident that we have conceived of a scenario S that provides us with good evidence for believing that S is possible?*

The answer is: *when we are confident that we have at least* ***not*** *violated the nature of the entities in question; that is, we have not violated what the fundamental nature of these entities is in our construction of the representations of the relevant scenarios*.

It might be objected that the PMEF (in this form) does not arise for Yablo’s conceivability-theory. Yablo’s theory does not claim that conceivability *entails* possibility. Instead it is content with the much weaker claim that conceivability *provides defeasible justification* for believing a possibility claim. According to Yablo (1993, p. 29), P is conceivable (for me) if I can imagine a scenario *that I take to* verify P. On this account the question that we take to generate the PMEF in this context seems to have a trivial answer: When should I be confident that I have conceived of a scenario *S* that provides me with good evidence for believing that *S* is possible?—Well, when (I am confident that) I take it to be so. Note, however, that this seemingly trivial answer does not render Yablo’s account immune to the PMEF. It rather defers the question on to the next level: When should I take it to be so? We take it that, here again, the following answer is plausible: *when I am confident that I have at least* ***not*** *violated the nature of the entities in question.*^7^ So, *S* counts as a scenario that a subject *x takes to verify* the possible existence of transparent iron based on what *x* knows about the essence of iron. Hence, Yablo’s conceivability-theory is susceptible to the PMEF.^8^

On *conceivability*-*theory* the epistemic friction for our believing that a conceived scenario is genuinely possible derives from our knowledge of what things are. To take note of an old idea from Arnauld’s exchange with Descartes: conceivability cannot be a reliable guide to what is really possible, if it is based on *ignorance* of *what* things are. Formal possibility might not track real possibility.

Let’s now turn to counterfactual-theory. In developing the PMEF in that case we will show that counterfactual-theory and conceivability-theory share a common problem with respect to epistemic friction.

## Counterfactual-theory

Williamson (2007) articulates and defends a counterfactual-theory of the epistemology of modality. Williamson’s account rests on the claim that metaphysical modality reduces to counterfactual conditionals at the metaphysical level.

(□): □P ≡_eq_ (¬P $${\square}\!\!\rightarrow$$ ⊥)
  The necessary is that whose negation *would* imply a contradiction.
(◊): ◊P ≡_eq_ ¬(P $${\square}\!\!\rightarrow$$ ⊥)
  The possible is that which *would* not imply a contradiction.

From this he concludes that the epistemology of modality is nothing but a special case of the epistemology of counterfactuals.^9^ Let’s look at the example Williamson gives to elucidate his epistemology:

(R): If the bush had not been there the rock would have ended in the lake.

In order to evaluate (R)

i. we *suppose* the antecedent (the absence of the bush) either by perceptually or non-perceptually *imagining* that scenario; and
ii. we *develop* the supposition, adding further judgments within the supposition by *reasoning*, *offline predicative mechanisms*, and other offline judgments—i.e. our *background knowledge and beliefs* (Williamson 2007, pp. 152f).

If the counterfactual development (CD) of the (supposed) antecedent eventually leads us to adding the consequent, we assent to the counterfactual. If it doesn’t lead us to adding the consequent (even after a robust CD), we dissent from it (Williamson 2007, pp. 152f).

Let’s now apply this to a case involving modality. According to (□) and (◊) modal claims are equivalent to counterfactual conditionals with a contradiction as their consequent or negations of them. So, you come to know that it is *necessary* that gold is the element with atomic number 79 by imaginatively supposing that gold is not the element with atomic number 79 and by counterfactually developing this supposition using reasoning, offline mechanisms, and background knowledge. If this CD leads us to a contradiction, we assent to the counterfactual and we thus come to know that it is *necessary* that gold is the element with atomic number 79.

Not only can it be shown that Williamson’s counterfactual-theory shares the PMEF with conceivability-theory, but it can also be shown that the very problem itself can be developed and seen in more detail by an examination of it within counterfactual-theory.^10^ According to the theory, for a CD to yield a contradiction, such as in the case of transparent iron, we have to restrain our offline mechanisms viz. our imagination by—as Williamson says—holding fixed some background information. This result is not surprising, since the counterfactual-theory at hand makes crucial use of exercises of imagination viz. conceivability.

Thus, our fallible imaginative evaluation of counterfactuals has a conceivability test for possibility and an inconceivability test for impossibility as fallible special cases. Such conceivability and inconceivability will be subject to the same constraints, whatever they are, as counterfactual conditionals in general, concerning which parts of our background information are held fixed. If we know enough chemistry, our counterfactual development of the supposition that gold is [not] the element with atomic number 79 will generate a contradiction. (Williamson 2007, pp. 164)

Let’s look at this step-by-step. The imaginative assumption that gold is not the element with atomic number 79 will generate a contradiction with the background information about chemistry that gold indeed is the element with atomic number 79.

**Table:** 

(1)	¬(Gold = E_79_)	(imaginative supposition)
(2)	Gold = E_79_	(background information held fixed)
∴		
(3)	⊥	[from (1) and (2)]

That is, the background information serves as a constraint on the imaginative exercise and as a regimentation of the CD. In other words, it is this very background information that creates the *epistemic friction* that is needed in CD to reliably yield modal knowledge. The crucial question in this connection is, however, *what* the *relevant* background information is, such that it needs to be held fixed. How do we know which background information can/must be held fixed? To see that this question is of importance, just consider a CD where (2) is not among the background information that is held fixed. We would not arrive at a contradiction and we would thus have to conclude that (1) is possible. Conversely, holding fixed, e.g., the information that gold is stored in Fort Knox would generate a contradiction with the supposition that it is not the case that gold is stored in Fort Knox, thereby yielding the false result that it is necessary that gold is stored in Fort Knox. It is of vital importance that the right or relevant background information is held fixed, so as to not under- or over- generate modal claims.^11^ Williamson is well aware of this so-called problem of *cotenablity*. He notes that some but not all of one’s background knowledge and beliefs are *cotenable* with the imagined supposition (Williamson 2007, pp. 152f). He emphasizes that we generally develop counterfactual suppositions by holding fixed *constitutive facts*, such as that gold is the element with atomic number 79 (Williamson 2007, p. 164).

So, in Williamson’s counterfactual-theory it is the constitutive, i.e. the *essentialist facts* about the objects which figure in the supposed scenarios that are cotenable with the supposition. These essentialist facts are the ones that *essentially* need to be held fixed in CD and that, as a consequence, provide the modal epistemic friction in the account. So, there is an analogical problematic structure in both conceivability-theories and counterfactual-theory. *Both kinds of theories require that some kind of epistemic friction be produced in some way by the very natures of the entities involved.* Both, conceivability exercises and CD, reliably track metaphysical possibilities *only if* the natures of the entities involved have not been violated in their course. As a consequence, one might argue that in order for one to be justified in believing that P is metaphysically possible, on the basis of conceiving of a scenario *S* or counterfactually reasoning and finding no contradiction, *it must be the case that they are justified in believing that they are not violating the natures of the entities involved.* We will return to a discussion of this last point after we identify where in *deduction*-*theory* one can find the PMEF.^12^

## Deduction-theory

Deduction-theory derives from the work of Kripke (1971). It can generally be discussed by looking at a basic pattern of inference that one can use for coming to know modal claims. The core inference pattern on the necessity-first approach is the following:^13^

**Table:** 

(4)	P → □P
(5)	P
∴	
(6)	□P

Vaidya (2017) notes that there are many critical questions one can ask about deduction-theories, given that we are pursuing a development of the PMEF, our interest here will be on the *justification question*: how are we justified in coming to know principles of the form ‘P → □P’? In the case of conceivability-theories and counterfactual-theory it is the *input question* that leads us to understanding and articulation of the PMEF. However, in the case of deduction-theory, the problem is generated via the *justification question*. If we want to deductively infer □P from P, we only get to do so, via the major premise (i.e. principles of the form) ‘P → □P’. In this sense, it is the latter principles that can be said to account for the epistemic friction needed to inferentially transition from P to □P. So, the crucial questions about epistemic friction, with regard to deduction-theory, are: what instances of ‘P → □P’ hold? And: how do we know them?

Since our concern here is with metaphysical modality, the metaphysical part of the question is: what is metaphysical modality? It was Kripke (1980) who introduced us to a use of ‘metaphysical modality’, and he did so through the use of examples, such as the necessity of identity or the necessity of origin. Hence, the PMEF for Kripkean deduction-theory would center around the question of how we know these and potentially other principles that govern metaphysical modality.

However, we need not stop with Kripke’s story. Staying within the realist camp, there is an alternative answer to the metaphysical question: what is metaphysical modality? It comes from the work of Fine (1994). On this account metaphysical necessity is just the kind of necessity that is grounded in the *essences* or natures of things. One could interpret this account as holding that metaphysical necessity is not determined by a variety of principles but by a manifold of instantiations of one single general principle of the following form:

(E): □_*x*_P → □P
  If a proposition P is true in virtue of the essence of some object(s) *x*, P is metaphysically necessary.^14^

In a way, (E) answers the initial question of what instances of ‘P → □P’ do in fact hold: whenever P is an essentialist truth, P is metaphysically necessary.^15^ It is important to note that the essentialist answer to the metaphysical question of what governs metaphysical modality is, in a sense, generic. It tells us that metaphysical modality is grounded in the natures or essences of things. It is generic in the sense that it does not tell us what pertains to the essences of things. This is important for the PMEF. An essentialist version of deduction-theory takes the following form:

**Table:** 

(7)	□_*x*_P → □P
(8)	□_*x*_P
∴	
(9)	□P

We take it that in order for us to be justified in believing a modal proposition on the basis of *essentialist deduction*-*theory*, we have to be justified in believing both the premises from which we infer the modal proposition.^16^ This means that we have to be justified in believing the link between essences and modality (i.e. principle (E), the major premise) *and* that some specific proposition belongs to the essence of some entity in question (i.e. the minor premise). Put in terms of the PMEF, what provides epistemic friction in essentialist deduction-theory is the general principle (E), *together* with the information about what the instances of (E) are.

Take the following example: Suppose you know that Socrates is human. To get from there to the knowledge that it is metaphysically necessary that Socrates is human on the basis of essentialist deduction-theory, what you need to know is that being human is *essential* to Socrates and that what is essential (to some *x*) is metaphysically necessary. In other words, the *epistemic friction creator* (EFC) in this case is the *essentialist principle* (E) *together* with some *essentialist proposition* (‘□_*x*_P’).

## The problem of modal epistemic friction (PMEF)

Let’s take stock: Sects. 2 and 3 have shown that conceivability-theories and counterfactual-theory face the PMEF through the need to supplement the process of conceiving, imagining and counterfactually developing an imagined supposition with constitutive/essentialist facts in order to make the respective process reliable. That is, were we to conceive of a scenario or develop a counterfactual supposition concerning an entity *x* on the basis of a false conception of what *x* essentially is, we ought not to think that our judgment is reliable, given that it is *based* on a misconception of what *x* is. In Sect. 4 we argued that deduction-theories face the PMEF through the need to supply justification for principles of the form ‘P → □P’ viz. ‘□_*x*_P → □P’, i.e. the major premise in the (respective) deduction model. If we have little to no justification for believing some conditional of the form ‘P → □P’ (or: ‘□_*x*_P → □P’), then why should we be justified in believing □P on the basis of deducing it from a justified belief that P (or: that □_*x*_P)? Typically, a person is justified in believing the conclusion of a deduction on the basis of inferring it correctly from the premise(s), which one also has justification for.

Generalizing, it can be said that the challenge the PMEF poses is twofold. It can be voiced in two questions that have to be answered by any account facing the PMEF:

**Table:** 

(PMEF)	(i)	What is it that creates modal epistemic friction (the EFC) in an account of modal knowledge?
	(ii)	How do we know that which creates modal epistemic friction (the EFC)?

Our discussions in Sects. 2–4 already gave an answer to (i): That which creates modal epistemic friction in conceivability-theories and in counterfactual-theory are *essentialist propositions*. If we take a Finean position in the metaphysics of modality—as we do in this paper—that which creates epistemic friction in deduction-theory are also *essentialist propositions* together with *essentialist principles*.^17^

The second part of (PMEF) derives from the idea that we have to know that which creates epistemic friction *in order* for it to create epistemic friction. In other words, on this picture, the relevant EFC is some part of our background knowledge against which we can check exercises of *conceivability*, properly develop a *counterfactual* supposition, or *conclude* that something is necessary or possible.

Given that the PMEF imposes this twofold challenge, an essentialist answer to (i) entails that we need to know essentialist propositions and principles in order to get to modal knowledge on these accounts. This leads naturally to *the epistemology of essence*.

## Do we need to know that which creates epistemic friction?

Before we can move to a discussion of how the epistemology of essence fits into the epistemology of modality (Sect. 7), we need to first discuss a possible criticism of the PMEF that attacks its second part by denying that *we have to know* the EFCs in the first place.

Williamson (2007) can be taken to argue that we do not need to know that which creates epistemic friction in the first place. In Sect. 3 we showed that the background beliefs cotenable with the imaginative supposition in a counterfactual development (CD) are the epistemic friction creators in Williamson’s account. Going back to the initial example about the bush and the rock, Williamson (2007, p. 143) argues that our sense of how nature works constrains our imaginative exercise such that it proceeds as “realistically” as it can. That is, the cotenable background information is, as Williamson puts it, our *folk physics*. Williamson has it, though, that folk physics is—though reliable enough to use in the acquisition of knowledge—strictly speaking *false*. Thus, by the factivity of knowledge, folk physics cannot be known. If in this case it is impossible for that which creates epistemic friction to be known, then it seems as if it is not necessary to *know* that which creates epistemic friction for it to create epistemic friction.

What would this mean for the PMEF? Remember that the problem consists of two controlling questions: (i) what is it that creates epistemic friction? And: (ii) how do we know that which creates epistemic friction? If Williamson is right and the epistemic friction creator does not need to be known, he can easily fend off the worry about epistemic friction. Note that (i) would be plausibly answered by the descriptive observation that “projecting *constitutive matters* […] into counterfactual suppositions is part of our general way of assessing counterfactuals” (Williamson 2007, p. 170; *emphasis added*) and (ii) would no longer be pressing.

The PMEF, however, re-arises in a different form. Williamson (2007, p. 145) argues that a subject undertaking a CD can rely on her folk physics to create epistemic friction, even if her folk physics is stored in a way, such that she is unable to articulate central parts of it in propositional form.^18^ Yet, it seems to be plain that her folk physics needs to be “stored” in some way whatsoever to be able to create epistemic friction. Hence, while for a specific domain the class of epistemic friction creators might be innate, generally that which creates epistemic friction needs to be epistemically acquired, i.e. we need to have some form of epistemic access to the EFC, even if this need not amount to knowledge. So, the PMEF re-arises in a slightly different form. We still need to know: (i) what it is that creates epistemic friction? And (ii*): how do we acquire *epistemic access* to that which creates epistemic friction?

The EFCs in Williamson’s counterfactual-theory are background beliefs cotenable with the imagined supposition. So, clearly the epistemic subject needs some form of epistemic access to these cotenable beliefs to create epistemic friction with the imagined supposition. Suppose the EFC in a given CD is the folk physics of a subject. The claim here is trivial: The subject has to *have* a folk theory, i.e. she must have acquired the relevant information for epistemic friction to be created. The consequence is that the acquisition of the relevant background information cotenable with the imagined supposition must be part of the story in the epistemology of modality.

So, it is safe to say that in order to be able to carry out a given CD, we must have access to the (web of) beliefs cotenable with the imagined supposition under consideration.^19^ But what exactly does it mean to have access to the cotenable beliefs? Does it suffice to be acquainted with what is cotenable or do we additionally need to understand *that* what we are acquainted with is cotenable with the imagined supposition? There is a crucial difference between (a) knowing (or having epistemic access to) some fact *f*, which is cotenable with the imagined supposition and (b) knowing (or having epistemic access to the fact) *that f* is cotenable with the imagined supposition.^20^

The examples revolving around gold’s atomic number and its being stored in Fort Knox in Sect. 3 have shown that the success of a CD crucially depends on whether or not the *right* background beliefs are held fixed. So, it seems that the epistemic subject—in addition to having access to the cotenable beliefs—must be able to know the cotenable ones from the ones that are not to be held fixed. Williamson, however, would want to deny this. To see why, it is best to assume an initially plausible cotenability criterion and look at a rough version of a prominent objection against Williamson’s counterfactual-theory as well as his reply to the objection.

One initially plausible cotenability criterion is the following: all facts that hold with *metaphysical necessity* should be held fixed in a CD of any imagined supposition whatsoever. This criterion is initially plausible, since the facts that are to be held fixed are plausibly those that cannot be varied. Since no metaphysically necessary fact can be varied, it is plausible to hold fixed all the facts that hold with metaphysical necessity.^21^ This, however, would raise the following objection: If the epistemic subject would have to know the cotenability status (i.e. in this case the *modal* status) of a fact in order for it to be held fixed and to create epistemic friction, Williamson’s CD would *presuppose* knowledge of metaphysical modality. As a consequence, CD would be *ill fitted* to be the single basic method for providing knowledge of metaphysical modality.^22^ Here is what Williamson offers as reply to this kind of objection:

[W]e need not judge that it is metaphysically necessary that gold is the element with atomic number 79 *before* invoking the proposition that gold is the element with atomic number 79 in the development of a counterfactual supposition. Rather, projecting constitutive matters such as atomic numbers into counterfactual suppositions is part of our general way of assessing counterfactuals. (Williamson 2007, p. 170)

This rejoinder can be interpreted in two ways: First, we might read this passage as saying that we should *drop the requirement* that the epistemic subject has to know (or even judge) that a cotenable belief is in fact cotenable for it to be held fixed and to create epistemic friction, since ordinary epistemic subjects are usually reliable in holding fixed the right, i.e. the cotenable ones, without being able to tell them apart from the non-cotenable ones. Second, it might be read as a *rejection* of the *initially* plausible *cotenability* criterion according to which we should hold the *necessary* facts fixed, since it is part of our general way of assessing counterfactuals to hold the *constitutive* facts fixed. By the end of Sect. 3 we took the constitutive facts to be the essentialist facts. Obviously, essentialist facts are metaphysically necessary. Still, the above cotenability criterion might be rejected by emphasizing the point about constitutive facts, since it might be that we should hold fixed the constitutive facts *not in virtue of them being necessary but in virtue of them being constitutive, i.e. essential.* We will come back to this thought in Sect. 7. For now, let us turn to the first interpretation of Williamson’s rejoinder.

Yli-Vakkuri (2013) pursues a counterfactual account that is quite different from Williamson’s project (in as much as it does not rely on CD).^23^ We are not taking issue with his original view. However, he does at (2013, pp. 618f) offer us a version of the first interpretation of Williamson’s work that is highly instructive to engage with for our concern. According to the first interpretation the subject does not need to *know* the cotenable facts in a distinguishable way from those that are not cotenable with the imagined supposition. It suffices that she is in some sense *sensitive* to the right ones. Yet, what does it mean that the subject is appropriately sensitive to the right ones? There is an externalist (EXT) and an internalist (INT) interpretation to be had.

(EXT): Ordinary subjects just happen to be *reliable* in holding fixed the right facts.
(INT): Ordinary subjects have some *epistemic access* (short of knowledge) to some criterion as to which facts are the right facts to be held fixed.

Yli-Vakkuri and Williamson clearly mean something along the lines of (EXT). One rationale for (EXT) can be accessed by looking at Yli-Vakkuri’s (2013, pp. 618f) criticism of Roca-Royes’ objection against Williamson. Roca-Royes (2011a, p. 38) argues that in order for Williamson’s CD to be knowledge-conducive we must *knowledgeably* hold the cotenable background beliefs fixed.^24^ Yli-Vakkuri (2013, p. 619) replies as follows:

Roca-Royes, for her part, makes use of the premise that ‘‘if our counterfactual judgments are to amount to counterfactual knowledge, it cannot be a matter of chance that we just *happen* to hold fixed (the) constitutive facts’’, and moreover we ‘‘must *knowledgeably* hold them fixed’’ (38, original emphases). This is an implausible requirement, as we can see by considering an analogy with perception. For your judgment that the patch of grass is green to count as knowledge, it is not sufficient that your visual system just by *chance* presents you with a green experience when you glance at the grass—that much is true. But it would be highly implausible to say that you do not know that the patch of grass is green unless you are knowledgeably sensitive to the right wavelengths. Something short of knowledge is enough: e.g., for a reliabilist it suffices that your visual system is reliably sensitive to the appropriate wavelengths. (Yli-Vakkuri 2013, p. 619)

Yli-Vakkuri builds his case against Roca-Royes’ internalist claim through (a) an analogy between CD and perception and (b) a reliabilist/externalist interpretation of perception. However, while the analogy draws out a very important point for considering the plausibility of the internalist position, it is not convincing in a crucial respect. In order to avoid the result that my visual system by *chance* presents me with a green experience, my visual system must be appropriately sensitive to the right wavelengths, such that the appropriate causal connection between my experience and the patch of green grass can be in place. It is, however, not in my power as an epistemic subject to put this appropriate causal connection in place, nor is it in my power to care for the appropriate sensitivity of my visual system to the right wavelengths. It seems the epistemic subject can’t do anything to establish the reliable link between her visual system’s presentation of a green experience and the right wavelengths. It is thus plausible that the epistemic subject does not need to be aware of, let alone know about, this sensitivity. Yet, contrary to the perception case, it is plausible that an epistemic subject performing a CD is able to establish the sensitivity to the right background beliefs. The subject performing a CD can (or could) do a great deal to establish that all, or at least some, of the right background beliefs are held fixed. Having access to some cotenability criterion would do the trick. Simply put, one might say that there is an *asymmetry in available epistemic agency between perception and imagination in CD*, even if one distinguishes between different kinds of imagination.

The fact that it does not lie in a subject’s power—and therefore not in her epistemic responsibility—to establish a (reliable) relation between her experiences and their *causes* does *not* entail the claim that it does *not* lie in the subject’s epistemic responsibility to establish a (reliable) relation to her EFCs, which can be taken as parts of the subject’s *evidence*. Put in slogan form: It does not follow from the fact that *we need not* be aware of the *causes* of our experiences that we do not need to be aware of our *evidence*, nor does it mean that we are not responsible for appealing to a source of evidence that is reliable.

To be sure, there might be different arguments for the claim that we need not be aware of a cotenability criterion in a CD. We find this argument by analogy unconvincing. We do not mean to suggest that this is the last word in the debate between an internalist and an externalist interpretation of what it means to be appropriately sensitive to an epistemic friction creator. Settling this debate is far beyond the scope of this paper. We invite further debate and examination.

What we have shown is that the problem of modal epistemic friction is *not only* a problem for the *internalist*. Our reasons were twofold. First, question (i) “what is it that creates epistemic friction?” is still crucial for the *externalist*. The externalist still has to tell a story as to what the parameters are of a reliable belief forming process. If a reliable belief forming process needs EFCs, the externalist’s story should at least give an answer to (i) and tell us what the EFCs for the reliable belief forming processes in question are. Second, even though the externalist rejects that the epistemic subject needs to have access to a cotenability criterion, i.e. that the epistemic subject needs to be able to tell *that* she is acquainted with an EFC in addition to being merely acquainted with an EFC, the reliabilist cannot (easily) reject that the subject must somehow be acquainted with the EFC to create epistemic friction. So, both parts of the problem are present for the externalist, even though externalism renders the second part of the PMEF [i.e. question (ii)] less forceful. In exchange for that, however, the externalist—as we have shown—has some more work to do to support the claim that it is not in the epistemic subject’s own responsibility to *make* the belief forming process (i.e. CD) reliable by holding fixed all and only those facts that are cotenable with the imagined supposition in question (through applying some accessible cotenability criterion). Holding fixed the right set cannot be magic.^25^

Summing up, the conclusion we drew from the PMEF in Sect. 5, that we need to *know* essentialist propositions and principles in order to get to modal knowledge, has to be weakened in light of Williamson’s point that our acquaintance with the EFCs might fall short of *knowledge*. What is still true, however, is that we need to have *some epistemic access* to essentialist propositions and principles in order to have epistemic access to modal propositions on the accounts discussed. Externalist arguments, if successful, might cast doubt on whether we need to be aware *that* the essentialist propositions acting as EFCs are in fact *essentialist* truths and do in fact act as EFCs. Externalism cannot, however, make us doubtful about the fact that these propositions need to be at our disposal in order to do their job as EFCs. A plausible externalist account in the epistemology of modality would neither escape the PMEF nor the conclusion that we need epistemic access to essentialist propositions. Yet it would render the PMEF less forceful, for it would probably escape the conclusion that the epistemology of essence is a *necessary* precondition for the epistemology of modality, since on the externalist picture we do not need to *judge* whether a proposition is essential, contrary to internalism. But, regardless of whether we are internalists or externalists, the PMEF shows that on all of the three accounts we discussed, a precondition for knowledge of metaphysical modality is that we have *access* to propositions that hold in virtue of the nature or essence of some object(s).

## Reductive and non-reductive Finean essentialism

So, if one is *not* inclined to take the externalist route, then it seems that the epistemology of metaphysical modality is intimately linked to the epistemology of essence. The PMEF shows that some epistemic access to essentialist propositions and principles has to precede our knowledge of metaphysical modality that is brought forth via any of the three accounts. Thus, no account in the epistemology of modality that involves any of these three methods is complete without telling a story as to how we acquire the needed essentialist information. This point, or a closely related one, is established by Roca-Royes (2011b).

Thus, as we see it the most pressing question in the epistemology of modality is the *essence question*: how do we come to know essences? Returning to the beginning of our exploration, we would add that the prospects for a possibility-first approach depends in part on how good of an answer can be given to the question concerning essence. Were it to turn out that no good answer to the essence question can be given, the plausibility of possibility-first approaches would seem to be increased on the assumption that one must take either an essence-first approach or a possibility-first approach. However, an answer to the *essence question* is beyond the scope of the current exploration. Our hope is that we have defended the view that future work should debate directly the relation between essence-first and possibility-first approaches to the epistemology of modality. Thus, in the remainder of our exploration we wish to tackle a different, ultimately *meta*-epistemological, question: what does it mean to say that essences are the *metaphysical* and *epistemological source* of metaphysical modality? In what follows, we show that the way one answers this question will affect the architecture of modal knowledge on the essentialist picture. We find our exploration here very useful for others also exploring essence-first approaches whether on the *Hale*-*Branch* or a variant of it.

Let’s start with the metaphysical side of the issue: what does it mean to hold that essences are the *metaphysical source* of metaphysical modality? Finean essentialism has it that metaphysically necessary truths obtain *because* essentialist truths obtain, the former hold *in virtue of* the latter. This can be expressed by the following grounding-claim that lies at the heart of Finean essentialism.

(FE): Essentialist truths *ground* metaphysically necessary truths.

On Fine’s neo-Aristotelian understanding, the essence of a thing *x* is the innermost core of *what x is*. As such, essences do not reduce to modality, as Fine’s (1994) counterexamples to the modal interpretation of essence have shown.^26^ One interesting question, however, is whether this neo-Aristotelian brand of essentialism is committed to the converse, i.e. that metaphysical modality reduces to essences. Some philosophers endorse such a reductive understanding of essentialism.^27^ It is not, however, clear what such a reduction amounts to. It is not plausible that being reduced to essences *eliminates* modality. Remember, (FE) says that modality is *grounded* in essence and that which is grounded *is* indeed less fundamental, yet *not* less *real* than its ground (cf. Schaffer 2009, Audi 2012, p. 102). Put differently: since grounding is *factive*, (FE) blocks such an eliminativist interpretation of the claim that modality reduces to essence.

Reducing modality to essence might amount to the claim that there is *no metaphysical gap* between essence and modality. Looking at some recent discussions of essentialism, one motivation for endorsing such a reductive thesis might be derived from epistemological concerns. Take essentialist deduction-theory. In Sect. 4 we saw that the EFCs in essentialist deduction-theory are twofold: In addition to having access to essentialist propositions, we need to be acquainted with an essentialist principle like (E), according to which any proposition that is true in virtue of the essence of some object(s) is also metaphysically necessary. Horvath (2014) can be said to have voiced a version of the PMEF for Lowe’s essentialist deduction-theory. He captures this point by noticing an *epistemic gap* between our knowledge of essentialist truths and our knowledge of corresponding modal truths that has to be bridged by knowledge of (or acquaintance with) some “bridge principle” like (E).

In order to avoid the need for *knowledge* of (or acquaintance with) some bridge principle like (E) in essentialist deductions one might go in for a reductive interpretation of Finean essentialism. We take it that this is the core of Tahko’s (2017, pp. 34f) answer to Horvath (2014). The idea is that if modality reduces to essence on the metaphysical level, there is *no metaphysical gap* between essence and modality. Hence, there is no *epistemic* gap either. However, whether reduction on the metaphysical level entails reduction on the epistemological level is problematic. Given that logical equivalence is *not* sufficient for epistemic reduction, why would metaphysical reduction be *sufficient* for epistemic reduction? More importantly, the mere *truth* of the claim that modality reduces to essence, one might argue, does *not* suffice for an epistemic subject to come to know a modal proposition directly by knowing an essentialist proposition without making any use of some bridge principle. Rather, the subject has to *know* that this reduction is in place, or she has to at least have some *epistemic access* to this reduction claim. And arguably, knowing that modality reduces to essence would resolve itself into knowing that any essentialist proposition is necessary, i.e. into knowing some bridge principle like (E).^28^

So, the *no*-*metaphysical*-*gap*-*interpretation* of the claim that modality *reduces* to essence cannot be used to play down the PMEF for essentialist deduction-theory. The fact that there is no metaphysical gap between essence and modality cannot undermine the need for epistemic access to some bridge principle like (E) in essentialist deductions.^29^ We might offer an analogy from the philosophy of science. The mere fact that biological facts could be reduced to chemical facts rests on the identification of a bridge principle that can take us from one special science to another special science. The bridge principle would be what justifies one in inferring specific instances of the general reducibility of biological facts to chemical facts.

However, there is another interpretation of the claim that modality *reduces* to essence that shall take center stage in the final part of our paper. (FE) provides a *grounding*-*explanation* of modality in terms of essences. We might ask whether this explanation is a *reductive* or a *non*-*reductive* one. According to the interpretation of ‘reduction’ we have in mind, this leads to the question: is the notion of ‘essence’, which modality is grounded in, itself a modal notion? With regard to the two possible answers to this question, we can distinguish two positions, *Reductive Finean Essentialism* (RFE) and *Non*-*Reductive Finean Essentialism* (NRFE); both of which endorse (FE), i.e. that modality is grounded in essences.

(RFE): Essences are entirely non-modal.
(NRFE): Essences are in some sense modal. They belong to the *larger family of modality.*

One might wonder why the second position is called ‘Non-Reductive *Finean* Essentialism’ for it seems clear that at the very heart of *Finean* essentialism is the rejection of the modal account of essence. Note, however, that (NRFE) is *not* a return to the modal account of essences according to which some *x* has a property F essentially iff *x* has F in every possible world (in which *x* exists). (NRFE) accepts Fine’s (1994) counterexamples to the modal view and uses a neo-Aristotelian conception of essence as the *whatness* of an object, the innermost core of what a thing is. Contrary to (RFE), however, (NRFE) does not take essences to be entirely non-modal but to be modal in some sense, without being reducible to *de re* necessity as the modal account of essence has it. Essences, according to (NRFE), belong to the *wider family of modality* but are not, as Fine has shown, analyzable in terms of possible worlds.

The following passage where Fine (2007, p. 85) clarifies his intentions in his “Essence and Modality” leaves the doors wide open for a position like (NRFE):

In claiming that the notion of essence was not to be understood in modal terms I had in mind the familiar metaphysical modalities, i.e. the familiar notions of metaphysical necessity, metaphysical possibility and the like. […] [I]t was not my intention to argue against an account of essence in terms of any modal notions whatever. (Fine 2007, p. 85)

Hale (2013, p. 61 fn. 1) is even more confident and seems to explicitly subscribe to (NRFE) by holding that “the notions of essence and essential property, even if not definable in terms of *de re* necessity, are to be regarded as modal notions”.^30^ Vetter (2011) gives a non-reductive interpretation of what she calls “New Actualism”, a family of views in modal metaphysics under which Fine’s essentialism is subsumed. She thereby indirectly opts for (NRFE).^31^ Very recently, Mallozzi (2018) displays strong leanings towards (NRFE) in her defense of re-considering Kripke’s theory of metaphysical modality, and the importance of putting metaphysics first in the epistemology of modality.

We are now ready to see, how (NRFE) and (RFE) deliver slightly different pictures of the architecture of modal knowledge: If (NRFE) is true, essences are themselves modal. This makes the epistemology of essences not only a precondition for the epistemology of modality but *part and parcel* of modal epistemology. According to (NRFE), our epistemic access to essence (whatever it may look like) is to be regarded as the fundamental epistemic access point to the realm of modality. Since (NRFE) regards essences as belonging to the modal realm, epistemic access to essences *is* epistemic access to the modal realm. Contrary to that, (RFE) has it that essences do *not themselves* belong to the modal realm. So, while (RFE) still claims that the epistemology of essence is a necessary precondition for the epistemology of modality the former is *not itself part and parcel* of the latter. This is because on the (RFE) picture, knowing an essence *is not itself* knowing something modal (even though it’s a precondition for knowing something modal).

It is important to note that this architectural difference makes for a difference in the effect the PMEF has on (NRFE) and on (RFE). According to the PMEF, all three accounts we considered in Sects. 2–4 crucially *presuppose* essentialist information in the epistemology of modality. This means that none of these three accounts can be regarded as the fundamental epistemic access point to essences on pain of circularity or regress. Now, under the assumption that (NRFE) is true and essences belong to the modal realm, these theories must fail as answers to what we call the *access question*. They cannot serve as the fundamental access point to *modality*. Note that on (RFE), even though an epistemology of essence is still a necessary precondition for the three discussed accounts to reliably yield modal knowledge, these accounts can still be regarded as answers to the access question, even though they presuppose rather than generate essentialist knowledge. This is because on (RFE), essentialist knowledge does not count as *modal* knowledge.

The fact that on (NRFE) none of the discussed accounts is apt to answer the access question does not render these accounts toothless. There are still many interesting and important *navigation questions* that can be appropriately handled by them. That is to say that even if these accounts cannot by themselves give us access to the modal realm *from outside the modal realm*, they are still apt ways to reason from one kind of modality (essences) to another. Yet, even the verdict that the access question is off limits to these three accounts does not seem to be true on all versions of (NRFE). In what follows we discuss an argument for Hale’s version of (NRFE) and show that it is compatible with the ability of conceivability-, counterfactual-, and deduction-theory to answer the access question, even though these accounts presuppose essentialist knowledge. The argument we consider draws on work by Blackburn (1993), Cameron (2010), and—of course—Hale (2002, 2013).

Blackburn (1993, p. 53) raises the following dilemma for any answer to the *question of what the source of necessity is*.

Suppose an eventual answer cites some truth F, and so takes the form: ‘A because F’. (‘Because’ here is taken to include constitutive variants: the truth that A consists in F, is made so by F, etc.) Now, either F will claim just that something is so, or it will claim that something must be so. If the latter, there is no problem about the form of the explanation, for one necessity can well explain another. But, as we have seen, there will be the same bad residual ‘must’: the advance will be representable as ‘if we see why this must be so, we can now see why that must be as well’. And there is no escape from the overall problem that way. Suppose instead that F just cites that something is so. If whatever it is does not have to be so, then there is strong pressure to feel that the original necessity has not been explained or identified, so much as undermined.

The dilemma takes the following form:

(B1): In an explanation of the source of necessity ‘□A because F’, the *explanans*, F, can either be contingent or necessary.
(B2): *Necessity Horn*: If F is necessary, necessity will not be explained, because the explanation would appeal to a necessity to explain a necessity.
(B3): *Contingency Horn*: If F is contingent, necessity will not be explained but undermined.

Cameron (2010) has shown that if we accept S4, we have to opt for the *Necessity Horn*.^32^ On the *Necessity Horn*, the source of a necessity must itself be necessary. (FE) takes the *source* (or *ground*) of modal propositions (□P) to lie in corresponding essentialist propositions (□_*x*_P). So, if we are to take the *Necessity Horn*, these essentialist propositions must themselves be necessary (□_*x*_P → □□_*x*_P).^33^ Yet, knowing what the *Necessity Horn* amounts to does not render it less bad. We still have the following situation: if the *explanans* needs to be necessary we presuppose what needs to be explained rather than explain it. So, one might wonder: is there a way out of Blackburn’s dilemma?

Hale’s (2002, p. 202) distinction between *transmissive* and *non*-*transmissive* explanations might provide a way to escape the dilemma. The *Necessity Horn* has it that whatever the *explanans* of the necessity of P is, it must itself be necessary. It is only in a *transmissive* explanation of the necessity of P, however, that the necessity of the *explanans* plays an explanatory role. In a *non*-*transmissive* explanation of the necessity of P, it is merely the truth of the *explanans* that explains the *explanandum*, not its necessity (even though the *explanans* might be indeed necessary).^34^ We can escape the dilemma by taking essentialist explanations of necessity to be non-transmissive explanations of necessity: For one, the *explanantia*—i.e. the essentialist propositions—are indeed necessary. Hence, there is, as Blackburn (1993, p. 53) puts it, “no problem about the form of the explanation, for one necessity can well explain another”. Yet, this does not generate the problem that Blackburn associates with the *Necessity Horn*, since the essentialist explanation of necessity is indeed *non*-*transmissive:* the necessity of the *explanans*, i.e. the necessity of the essentialist proposition does not play an explanatory role. Put differently, even though ‘□_*x*_P’ is indeed necessary, it is ‘□_*x*_P’ rather than ‘□□_*x*_P’ that explains ‘□P’. Thus, the essentialist explanation of modality (FE) does not presuppose what it seeks to explain.

Hale escapes the dilemma by arguing for the following two claims:

(α): The *explanans* in the essentialist explanation of necessity is indeed itself necessary. (*Necessity Horn*)
(β): The necessity of the *explanans* does not play a role in the essentialist explanation of necessity. (*Non*-*Transmissiveness*)

Hale takes (α) to be evidence enough for (NRFE), i.e. for the claim that the essentialist explanation of modality in (FE) is not reductive and that essences are themselves modal.

The point of the essentialist theory is not, then, to provide a reductive explanation of any necessities. It is, rather, to locate a base class of necessities—those which directly reflect the natures of things—in terms of which the remainder may be explained. The kind of explanation it offers, then, is not one which provides, as it were, an entry point into the class of necessities from outside—for no such ‘external’ explanation of necessity is possible—but one which exhibits the class of necessities as structured in a certain way, by identifying some necessities as basic or fundamental, and the rest as dependent, inheriting their necessity, ultimately, from necessities in the base class. (Hale 2013, pp. 158f)

So, on Hale’s view, what makes essentialities modal is that they are themselves necessarily true. Essentialist truths belong to the modal package in virtue of being themselves necessary. In other words, it is the Necessity Horn that makes Hale a proponent of (NRFE). Earlier we argued that on the (NRFE) picture, none of the three accounts discussed in Sects. 2–4 are fit to answer the access question. Yet, on Hale’s version of (NRFE), they are: Since essentialities *non*-*transmissively* explain necessities, it is plausible that the epistemic subject can be completely ignorant about the *necessity* of an essentiality and still be reliably guided to modal truth. Note further that since on Hale’s view essentialities are modal (only) in virtue of being necessities, no modal information is needed to be reliably guided to modal truth via essences. For short: that kind of essentialist information that is required in conceivability-, counterfactual-, and deduction-theory to reliably guide us to modal truth is not itself modal information on Hale’s account.

This result is surprising, since Hale (2013, p. 61 fn. 1) explicitly claims that “the notions of essence and essential property […] are to be regarded as modal notions”. So, what to make of this result? One might argue that Hale’s position that drops out of the argument from the Necessity Horn actually falls short of the claim that the notion of essence is a modal notion and, hence, does not truly qualify as a version of (NRFE). The mere fact that all essentialist propositions are themselves necessary does not make ‘essence’ a modal notion. In other words, even if it is true that for all propositions P, (□_*x*_P → □□_*x*_P) is true, it would not follow simply by asserting ‘□_*x*_P’ that I assert something modal. Hale’s argument shows that essentialist propositions are themselves necessary. Hence, they belong to the modal family, *in as much as they are necessities*. If every essentialist truth holds with necessity—i.e. if for all propositions P, (□_*x*_P → □□_*x*_P)—it comes as no surprise that essentialist truths belong to the modal realm. Yet, they do so in virtue of the necessity-operator (‘□’) and not in virtue of the essentiality-operator (‘□_*x*_’). One might, however, take (NRFE) to be the position that essentialities belong to the *wider package of modality in as much as they are essentialities*—i.e. in virtue of the essentiality-operator (‘□_*x*_’). Since this is not shown by Hale’s argument, there may be doubt as to whether his position should count as a version of (NRFE) in the first place.

If we take this line of reasoning seriously, a ‘proper’ version of (NRFE) would be one that takes essentialist propositions to be modal propositions, not because they are themselves necessary but simply in virtue of being what they are: *essentialist* propositions. What could an argument for such a version of (NRFE) look like? It would have to answer the question of why *essentiality*—i.e. that which grounds necessity—is to be considered a *modal* status and it would have to account for how exactly this modal status differs from necessity. We can neither provide such an argument nor such an account. Yet, we would like to close by pointing out where we think the search for such an argument could take its start: A possible motivation for such a ‘proper’ version of (NRFE) could be to think that just like it is problematic to derive a normative claim from entirely non-normative claims, it might be problematic to derive a modal proposition from entirely non-modal ones. On the assumption that essence is the metaphysical *and epistemic* ground of necessity, this thought motivates the claim that essences *qua essences* are modal. The decisive difficulty that this line of thought is faced with is the following, though: If it’s true that, just like we can’t have an *ought* from an *is*, we can’t derive something modal from something entirely non-modal, how could we ever answer the access question?

The purpose of this section was neither to argue for nor against Hale’s position.^35^ Nor did we strive to settle the debate between (RFE) and (NRFE). The goal was a more modest one: We wished to map out the distinction between (RFE) and (NRFE) and consider the respective effect the PMEF has on them. The fact that there might be doubt about whether Hale’s version of (NRFE) in fact is a version of (NRFE) indicates that there is more work to be done in mapping out the exact distinction between the possible positions as well as their motivations and the arguments in their support. Since this is beyond the scope of this paper, we leave this task to future research.

## Conclusion

Our investigation of conceivability-, counterfactual-, and deduction-theory led us to the claim that all three accounts rely on essentialist propositions and/or principles to create epistemic friction in order for them to reliably lead to modal knowledge. In light of our discussion of Williamson, Roca-Royes, and Yli-Vakkuri, we dropped the requirement that these essentialist propositions and/or principles, *epistemic friction creators*, need to be *known.* Instead, we argued for the weaker claim that we have to at least have *some epistemic access* to the EFCs in order for any of the accounts to reliably yield modal knowledge. We discussed an externalist rejection of the weaker claim and cast doubt on a specific externalist argument in favor of this rejection based on an analogy with perception. We finally moved to a discussion of what it means for essences to be the *metaphysical source* and *epistemological pathway* to metaphysical modality. In this course we introduced a distinction between two versions of essentialism: (RFE) and (NRFE), arguing that they deliver different pictures of the architecture of modal knowledge and that, consequently, the PMEF has a more serious impact on (NRFE) than on (RFE). Finally, we turned to an argument of Hale’s version of (NRFE). Assessing this argument gave us reason to either doubt that all versions of (NRFE) render conceivability-, counterfactual-, and deduction-theory inapt to answer the so-called access question, or to doubt that Hale’s position is a version of (NRFE) in the first place.

## Footnotes

1 For the purposes of this paper we will be presupposing the standard model of modality, known as the nesting model, on which there are at least three distinct kinds of modality: logical, metaphysical, and physical; where metaphysical possibility is a proper subset of logical possibility, and physical possibility is a proper subset of metaphysical possibility. While this model is not necessary for our investigation, it is useful to use the standard model in our investigation. More importantly, it should be noted that there are skeptics about the nesting model. *Inflationists* argue that metaphysical modality inflates so that it is co-extensive with logical modality. *Deflationists* argue that metaphysical modality deflates so that it is co-extensive with physical modality. Buzaglo (2010) provides an excellent discussion of an example from physics where we can see how physical modality comes apart from metaphysical modality in a way distinct from discussions typically focusing on theoretical identifications, such as the identity between Water and H_2_O. We, further, acknowledge that broad logical necessity *might be* coextensive with metaphysical necessity in the way Chalmers (2002) conceives it within the thesis of modal rationalism because broad, as opposed to narrow, will generally include conceptual truths; however, we maintain that narrow logical modality, modality about what can and cannot follow from the laws of logic alone cannot be coextensive with metaphysical modality. In addition, following Vaidya (2006), the laws of logic themselves are metaphysically necessary which shows that within the nesting model there are interesting relations between metaphysical modality and logical modality in that the former is a subset of the latter. Yet, the logical laws themselves have a special standing relative to metaphysics, namely that of metaphysical necessity. Even if logical pluralism holds, and there are a number of different ways of capturing what a valid argument is, it will still be true that pluralism is metaphysically necessary.

2 It is important to note that Hale’s classification admits of a third option: there is no dominant versus recessive relation in the epistemology of modality via necessity and possibility. On this view, the architecture of modal knowledge is messy and complicated. One way to work out this notion would be to hold that at least in some cases we are justified in believing it is possible for *a* to be F on the basis of holding fixed some essential truths about *a* and F as well as some possibility claims about *a* and F. For the purposes of this paper, though, we will be following the main distinction in Hale’s work.

3 That these three theories are the leading ones in the debate is in line with Strohminger and Yli-Vakkuri (2017). Note that what we call “deduction-theories” is subsumable under what they call “two-factor views”. Importantly, though, their distinction includes the work of Al Casullo, which falls under “two-factor views”, but will not be discussed here under our “deduction-theories”. Casullo (2003, 2010) offers a modest account of the relation between *a priority* and *modality*. We believe his view is important in the epistemology of modality because it defends a link between *a priority* and *modality*, we are forgoing discussion of it in order to draw attention to a problem that we are not confident applies to his work.

4 Although Peacocke’s (1998) theory of implicit knowledge is a contender in the epistemology of modality, we will forgo explicit engagement with it because of the problem we aim to develop. As Roca-Royes (2010) has already pointed out, Peacocke’s theory is directly open to the challenge that it rests on the epistemology of essence/constitutive knowledge.

5 The “epistemic transition” we are speaking of has been viewed historically to either be grounded in the (claimed) entailment between ◊^*c*^P and ◊^*x*^P—the entailment account of conceivability (Chalmers 2002)—or in the fact that ◊^*c*^P provides defeasible evidence/justification for (believing) ◊^*x*^P—the evidential account of conceivability (Yablo 1993). One crucial difference between Chalmers’ entailment account and Yablo’s evidential account lies in the kind of modality *x* that is involved in their respective claims. Importantly, and in addition, Roca-Royes (2011b) discusses a further difference between evidential and entailment accounts. She argues that the former is epistemic and that the latter is non-epistemic. This makes for a decisive difference in the kind of conceivability these accounts are working with.

6 Note that throughout the paper the term ‘the essence of *x*’ is used in the neo-Aristotelian or Finean sense, referring to the innermost core of what an object *x* is, i.e. the *whatness* of *x*—as opposed to the sense of essence according to which ‘essential’ and ‘necessary’ are synonymous. By the phrase ‘innermost core’ we don’t mean to connote anything spatial about essences, nor do we mean to connote a necessary connection to the distinction between intrinsic and extrinsic properties. See Sect. 7 for a discussion of Finean Essentialism.

7 A more powerful objection at this stage would be to hold that our requirement is *too demanding*. That to require a subject to only be confident when they have not violated the essences of the entities involved might seem to make justification for modal beliefs too difficult to acquire. Our view is that there must be a happy medium between the weak response, “whenever I take it to be so”, and the demanding response, “only when I know everything essential to the entities involved.” The way to see our claim here is that it would be odd for a subject to say, I know nothing about particle physics, but because I can imagine particle *a* with property F, I am justified in believing that it is possible for *a* to be F.

8 Thanks to an anonymous reviewer from *Synthese* for pushing us on this point.

9 For a critical discussion of this conclusion see Jenkins (2008) and Casullo (2012). For a defense of Williamson’s claim see Yli-Vakkuri (2013).

10 One might complain that the comparison between conceivability- and counterfactual-theory is asymmetric, since we are juxtaposing *generic* or *general* conceivability-theory and a *specific* (Williamsonian) counterfactual-theory. Note, however, that even though Williamson provides us with what looks like a *specific* account, it is—in an important sense—also generic. Williamson neither specifies (a) what kind of offline mechanism is at work, nor (b) what constitutes a robust enough CD, such that we can warrantedly assert ◊P on the condition that the CD of ¬P did not yield a contradiction. We do not, however, want to omit that there are counterfactual-theories that do not rely on CD. For discussion see Yli-Vakkuri (2013).

11 See also Roca-Royes (2011b) who makes a similar point.

12 See Sect. 6 for an evaluation of this point from an epistemic internalist and an epistemic externalist viewpoint, respectively.

13 Although Kripke (1971) introduces the deduction model for the case of a posteriori necessities, it is completely general. For example, one could come to know that a mathematical truth, such as that 2 + 2 = 4, holds of necessity through a set of premises all of which are known a priori. In addition, given the introduction, by Williamson (2007), of the notion of *armchair* knowledge as knowledge that is not strictly a priori or a posteriori, it is possible that one can come to know a modal truth through *armchair* reasoning.

14 We are using Fine’s subscripted box notation ‘□_*x*_P’ which reads: ‘P is true in virtue of the essence of *x*’.

15 See also Mallozzi (2018).

16 Arguably, this requires some commitment to a broadly internalist view. See Sect. 6 for a discussion of the PMEF from an epistemic internalist and an epistemic externalist viewpoint, respectively.

17 One might object that essentialist propositions (together with essentialist principles) cannot be all there is to epistemic friction. Consider a situation in which a subject *x* is well aware of what an object *a* is but *not* about what *a* could be. This situation appears to be one in which *x* has all the relevant essentialist information, yet not all the epistemic friction that is needed to restrict conceivability exercises, counterfactual development and inferential methods to acquire modal knowledge. Hence, there is more to epistemic friction than just essentialist information. Note, however, that any rational subject that has the relevant essentialist information about *what the objects in questions are* is in a position to know *what these objects could be*. This follows from the neo-Aristotelian, Finean picture of essences that we are working with in this paper. According to Finean Essentialism, essentialist information concerning the relevant objects in a scenario *grounds* (i.e. *determines*) the modal information about them. See Sect. 7 for a discussion of Finean Essentialism. We are indebted to an anonymous reviewer from *Synthese* for pushing us on this point.

18 From this he concludes that, even though explicit reasoning might be a part of it, CD cannot be reduced to explicit reasoning.

19 Note that for Williamson’s counterfactual-theory the problem of epistemic friction, *in a sense*, coincides with the cotenability problem: (i_c_) Which (web of) beliefs, propositions or facts (is) are cotenable with the imagined supposition? (ii*_c_) How do we acquire epistemic access to (this) these (web of) beliefs, propositions or facts?

20 Take for instance Luke Skywalker. Before the final showdown of *The Empire Strikes Back* he is (a) acquainted with (the person that is) his father, but he does *not* (yet) realize (b) *that* this person is his father.

21 Note that this also nicely agrees with the examples we gave: The proposition that gold = E_79_ is indeed metaphysically necessary, which is why (according to this criterion) it should be held fixed in a CD of *any* supposition (even of its negation).

22 Roca-Royes (2011a, b), Tahko (2012), and Casullo (2012) have all raised this objection in alternate ways.

23 See also fn. 10.

24 Note that Roca-Royes’ (internalist) claim is even stronger than (INT). It not only holds that the epistemic subject needs to have some sort of epistemic access to some cotenability criterion but also that this epistemic access must amount to knowledge.

25 What has to be conceded in favor of Yli-Vakkuri’s critique is that Roca-Royes’ claim *that for a CD to be knowledge-conducive the cotenable beliefs have to be held fixed knowledgably* seems *unnecessarily strong* (even from the perspective of internalism). In order to rule out pure chance, it suffices to claim that the cotenable beliefs need to be held fixed *justifiably*. Remember that this was also a result of our discussion in Sect. 3. We argued that in order for someone to be justified in believing that P is metaphysically possible (or necessary), on the basis of CD and finding no (or a) contradiction, it must be the case that they are *justified* in believing that in the course of this search no essentialist truth about the relevant objects involved are violated. Some externalists would claim that this latter justification stems from the fact that we ordinarily happen to *reliably* hold fixed the right background beliefs in a CD. Some internalists, on the other side, would want to argue that being justified in believing that no essentialist truth has been violated must involve some form of *epistemic access* to these essentialist truths.

26 Even though Socrates has the property of being a member of {Socrates} as a matter of metaphysical necessity, this property is not essential to Socrates, for it does not pertain to the innermost core of what Socrates is (Fine 1994, pp. 4f).

27 See e.g. Correia (2012) and Tahko (2017, 2018).

28 Note that all this presupposes that reducing modality to essence (as noted above) cannot entail the elimination of modality.

29 Note also that it is not clear whether one needs to hold that modality *reduces* to essences to claim that there is no metaphysical gap between essence and modality. For a discussion of the relation between grounding and reduction, see Rosen (2010) and Trogdon (2013). For a discussion of how grounding bridges metaphysical gaps, see Schaffer (2017).

30 Note that Hale does not seem to share our opinion on Fine’s openness to (NRFE): According to Hale (2013, p. 150 fn. 12) the major difference between his and Fine’s view is that “whereas Fine sees himself as giving a *reductive* explanation of modality in terms of essence or nature, I do not” (*emphasis added*). He thereby appeals to the difference between what we call (RFE) and (NRFE).

31 Note that Vetter (2011, p. 743) also explicitly states that essences belong to the “larger package of modality”. We borrow this notion from her, while sometimes interchanging *family* for *package*.

32 Due to space-limitations we will not rehearse the argument here. Suffice it to say that Cameron arrives at this conclusion by a *reductio* of the *Contingency Horn*. The only two controversial premises Cameron (2010, pp. 139f) uses in this *reductio* are the S4 principle (□P → □□P) and either one of the following principles (a) or (b): (a) if the source of necessity were lacking then the necessities *would* no longer be necessary; (b) if the source of necessity were lacking then the necessities *might* no longer be necessary. S4 is controversial. There are arguments against S4 due to Nathan Salmon (1989), which are re-articulated and further defended by Vaidya (2008) in the context of the epistemology of modality. However, there is a powerful response in favor of preserving S4 offered by Roca-Royes (2006) and a further defense in Williamson (2007).

33 Note that this (□_*x*_P → □□_*x*_P) is different from principle (E) (□_*x*_P → □P).

34 See also Cameron (2010, p. 144) for discussion.

35 Note that one dialectical weakness of Hale’s position could be seen in the fact that it presupposes S4 which has been widely acknowledged to be incompatible with Salmon’s (1982) *flexible origin essentialism*. Since many philosophers agree with flexible origin essentialism, Hale’s position might not be a live option for those philosophers. Note, however, that Williamson (1990), Hale (2013) and, very recently, Roca-Royes (2016) have argued for the compatibility of S4 and flexible origin essentialism. Thanks to an anonymous reviewer from *Synthese* for drawing our attention to this point.
